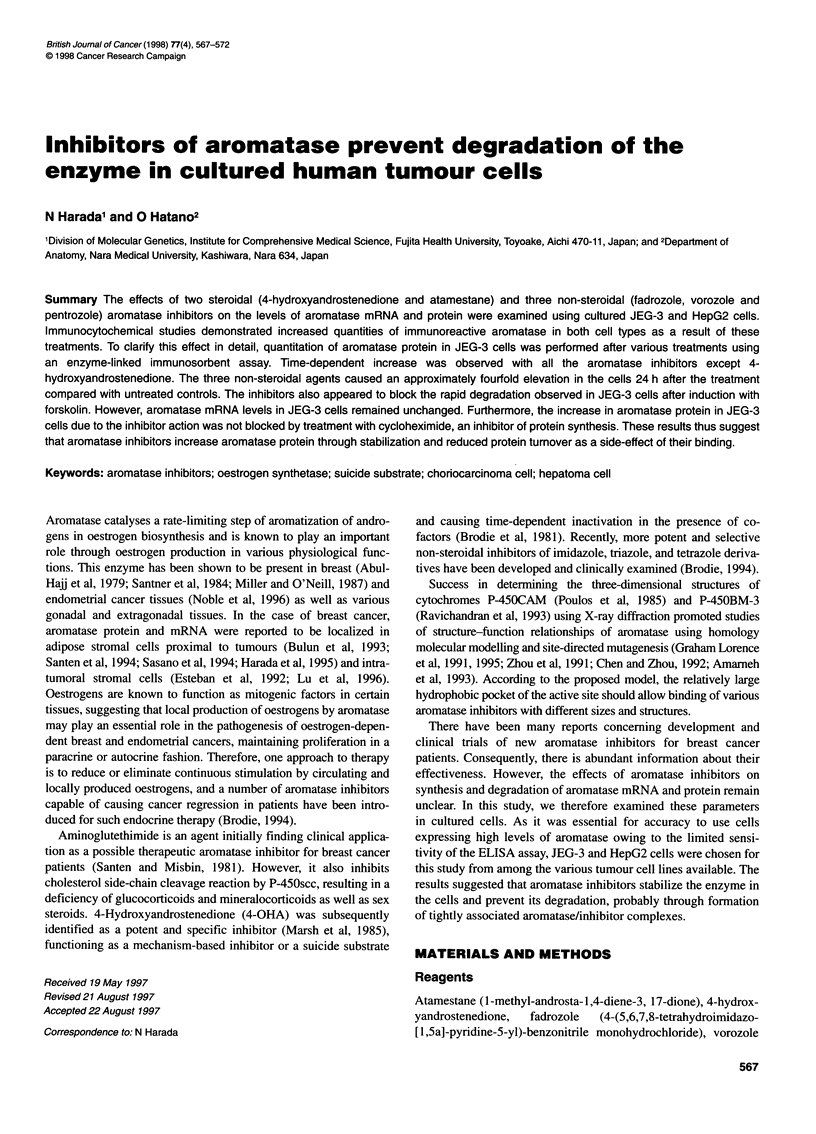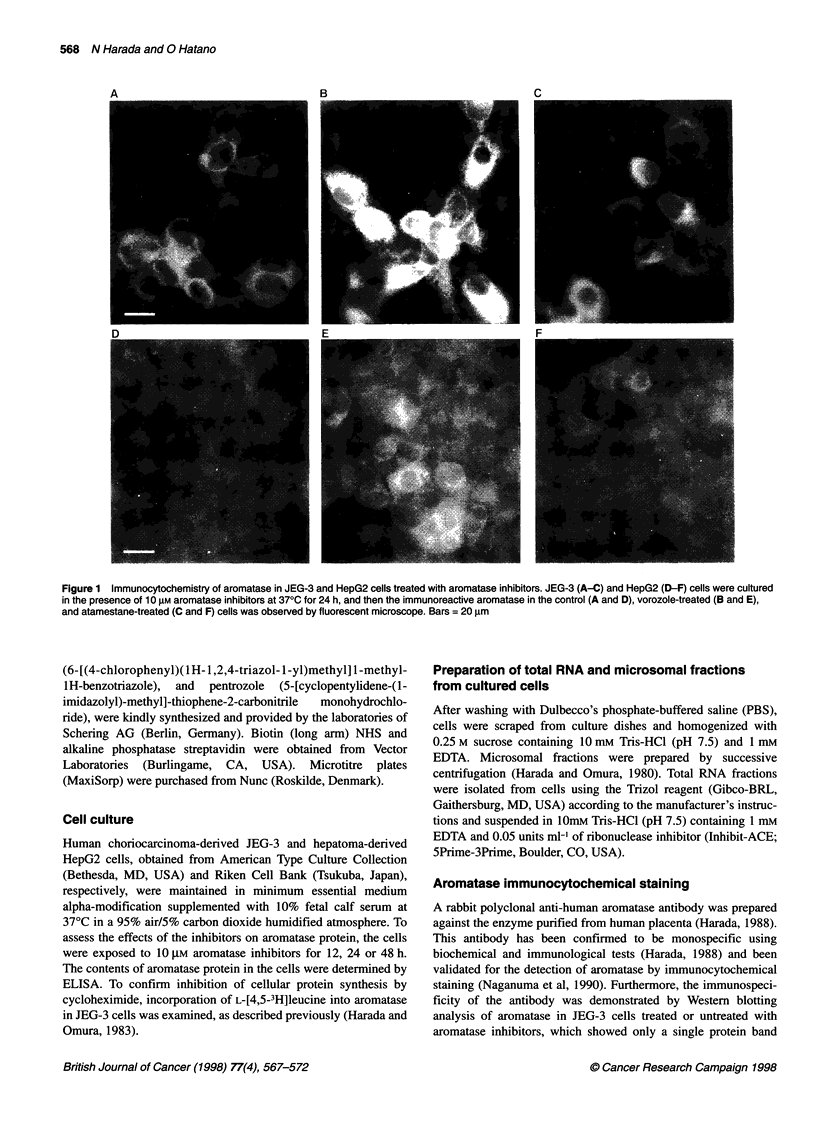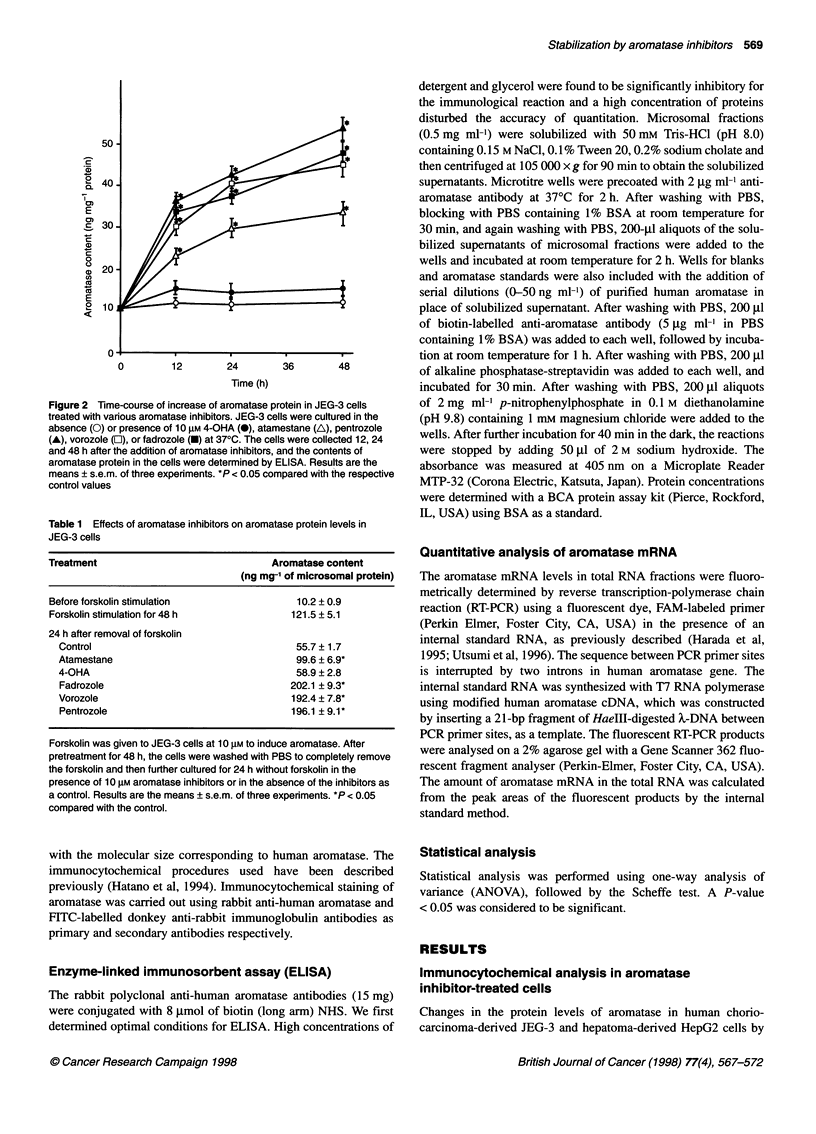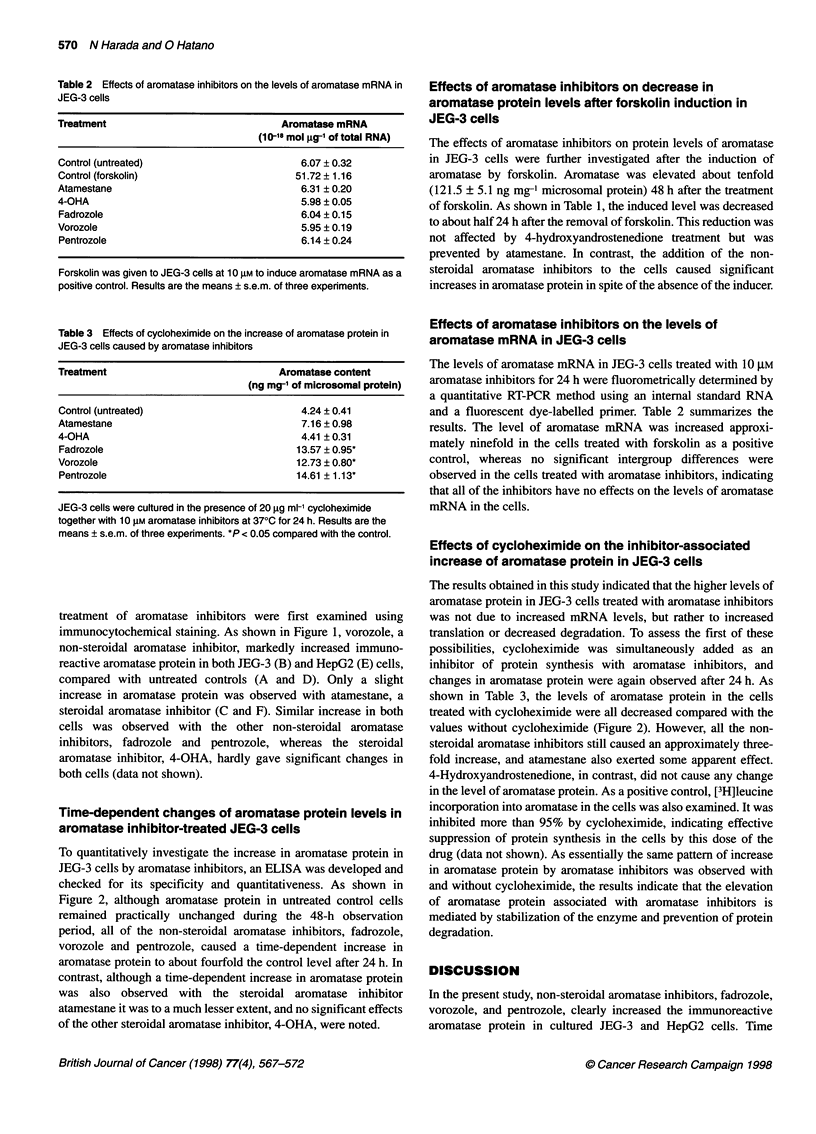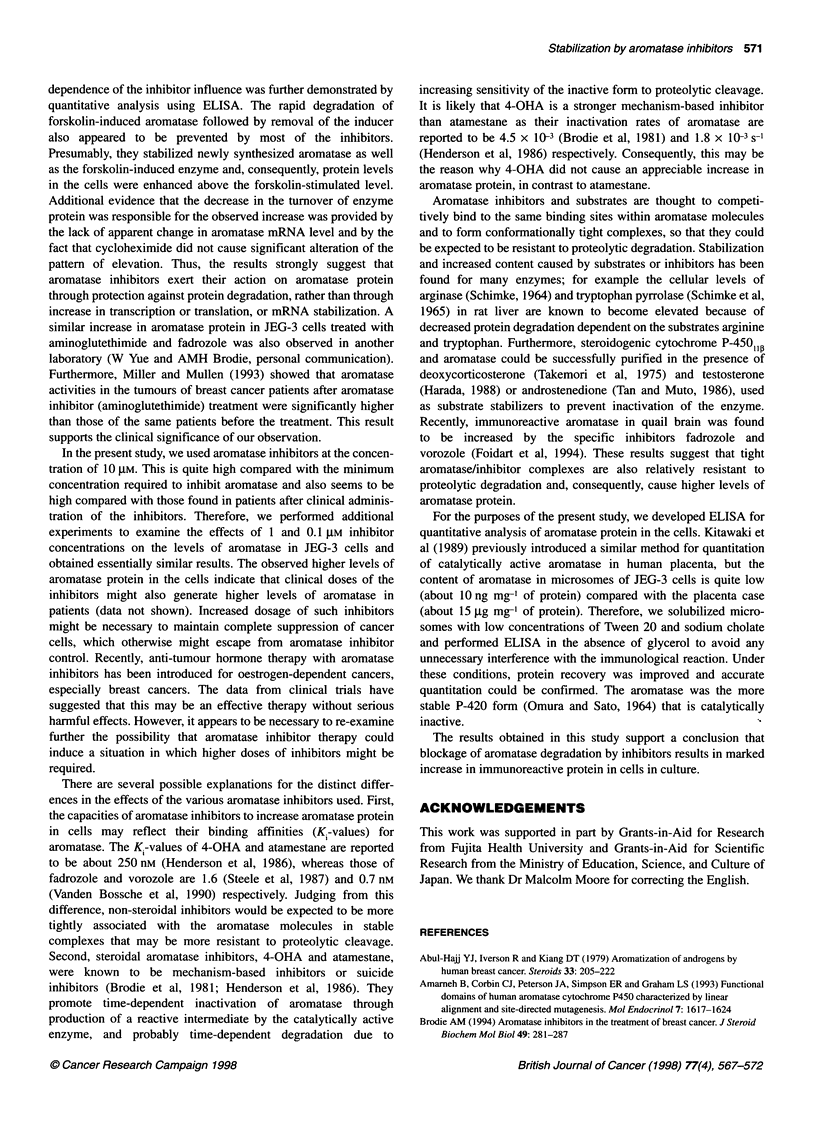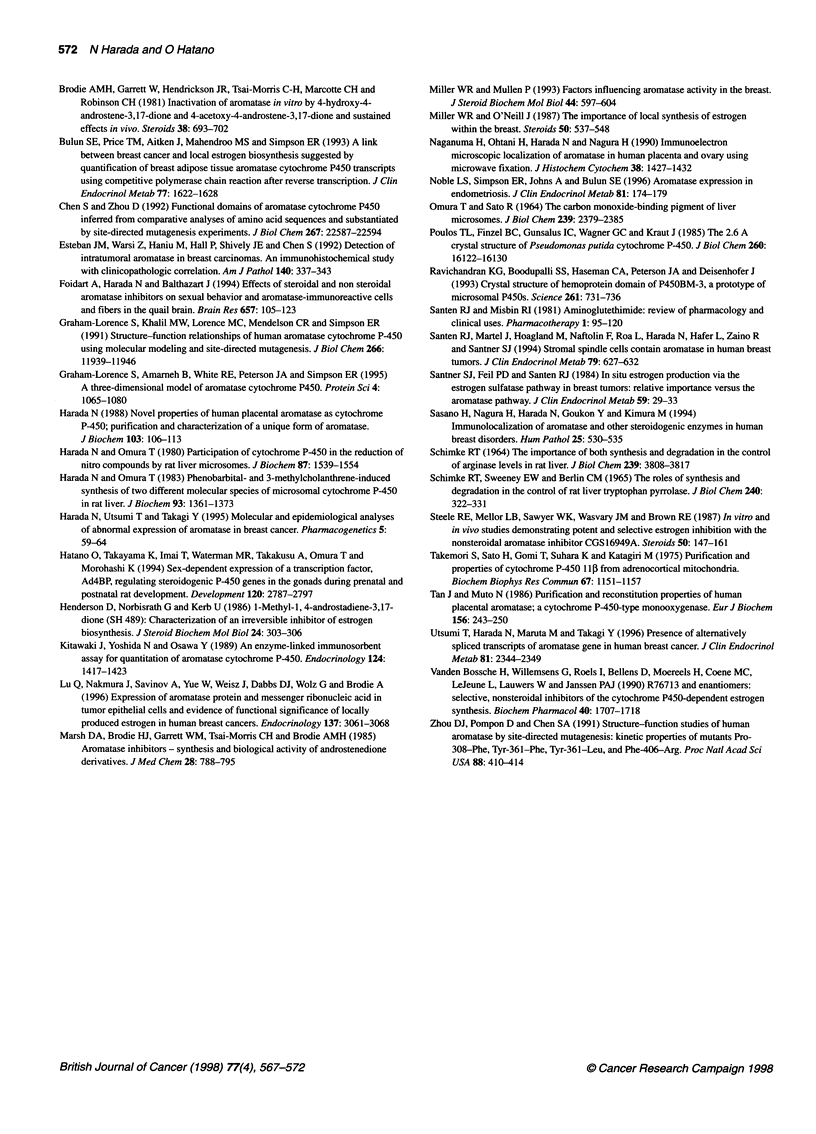# Inhibitors of aromatase prevent degradation of the enzyme in cultured human tumour cells.

**DOI:** 10.1038/bjc.1998.92

**Published:** 1998-02

**Authors:** N. Harada, O. Hatano

**Affiliations:** Division of Molecular Genetics, Institute for Comprehensive Medical Science, Fujita Health University, Toyoake, Aichi, Japan.

## Abstract

**Images:**


British Journal of Cancer (1998) 77(4), 567–572
© 1998 Cancer Research Campaign

### Inhibitors of aromatase prevent degradation of the enzyme in cultured human tumour cells

**N Harada[1] and O Hatano[2]**

[1]Division of Molecular Genetics, Institute for Comprehensive Medical Science, Fujita Health University, Toyoake, Aichi 470-11, Japan; and [2]Department of Anatomy, Nara Medical University, Kashiwara, Nara 634, Japan

**Summary** The effects of two steroidal (4-hydroxyandrostenedione and atamestane) and three non-steroidal (fadrozole, vorozole and pentrozole) aromatase inhibitors on the levels of aromatase mRNA and protein were examined using cultured JEG-3 and HepG2 cells. Immunocytochemical studies demonstrated increased quantities of immunoreactive aromatase in both cell types as a result of these treatments. To clarify this effect in detail, quantitation of aromatase protein in JEG-3 cells was performed after various treatments using an enzyme-linked immunosorbent assay. Time-dependent increase was observed with all the aromatase inhibitors except 4-hydroxyandrostenedione. The three non-steroidal agents caused an approximately fourfold elevation in the cells 24 h after the treatment compared with untreated controls. The inhibitors also appeared to block the rapid degradation observed in JEG-3 cells after induction with forskolin. However, aromatase mRNA levels in JEG-3 cells remained unchanged. Furthermore, the increase in aromatase protein in JEG-3 cells due to the inhibitor action was not blocked by treatment with cycloheximide, an inhibitor of protein synthesis. These results thus suggest that aromatase inhibitors increase aromatase protein through stabilization and reduced protein turnover as a side-effect of their binding.

**Keywords:** aromatase inhibitors; oestrogen synthetase; suicide substrate; choriocarcinoma cell; hepatoma cell

Aromatase catalyses a rate-limiting step of aromatization of androgens in oestrogen biosynthesis and is known to play an important role through oestrogen production in various physiological functions. This enzyme has been shown to be present in breast (Abul-Hajj et al, 1979; Santner et al, 1984; Miller and O'Neill, 1987) and endometrial cancer tissues (Noble et al, 1996) as well as various gonadal and extragonadal tissues. In the case of breast cancer, aromatase protein and mRNA were reported to be localized in adipose stromal cells proximal to tumours (Bulun et al, 1993; Santen et al, 1994; Sasano et al, 1994; Harada et al, 1995) and intratumoral stromal cells (Esteban et al, 1992; Lu et al, 1996). Oestrogens are known to function as mitogenic factors in certain tissues, suggesting that local production of oestrogens by aromatase may play an essential role in the pathogenesis of oestrogen-dependent breast and endometrial cancers, maintaining proliferation in a paracrine or autocrine fashion. Therefore, one approach to therapy is to reduce or eliminate continuous stimulation by circulating and locally produced oestrogens, and a number of aromatase inhibitors capable of causing cancer regression in patients have been introduced for such endocrine therapy (Brodie, 1994).

Aminoglutethimide is an agent initially finding clinical application as a possible therapeutic aromatase inhibitor for breast cancer patients (Santen and Misbin, 1981). However, it also inhibits cholesterol side-chain cleavage reaction by P-450scc, resulting in a deficiency of glucocorticoids and mineralocorticoids as well as sex steroids. 4-Hydroxyandrostenedione (4-OHA) was subsequently identified as a potent and specific inhibitor (Marsh et al, 1985), functioning as a mechanism-based inhibitor or a suicide substrate and causing time-dependent inactivation in the presence of cofactors (Brodie et al, 1981). Recently, more potent and selective non-steroidal inhibitors of imidazole, triazole, and tetrazole derivatives have been developed and clinically examined (Brodie, 1994).

Success in determining the three-dimensional structures of cytochromes P-450CAM (Poulos et al, 1985) and P-450BM-3 (Ravichandran et al, 1993) using X-ray diffraction promoted studies of structure–function relationships of aromatase using homology molecular modelling and site-directed mutagenesis (Graham Lorence et al, 1991, 1995; Zhou et al, 1991; Chen and Zhou, 1992; Amarneh et al, 1993). According to the proposed model, the relatively large hydrophobic pocket of the active site should allow binding of various aromatase inhibitors with different sizes and structures.

There have been many reports concerning development and clinical trials of new aromatase inhibitors for breast cancer patients. Consequently, there is abundant information about their effectiveness. However, the effects of aromatase inhibitors on synthesis and degradation of aromatase mRNA and protein remain unclear. In this study, we therefore examined these parameters in cultured cells. As it was essential for accuracy to use cells expressing high levels of aromatase owing to the limited sensitivity of the ELISA assay, JEG-3 and HepG2 cells were chosen for this study from among the various tumour cell lines available. The results suggested that aromatase inhibitors stabilize the enzyme in the cells and prevent its degradation, probably through formation of tightly associated aromatase/inhibitor complexes.

*Received 19 May 1997*
*Revised 21 August 1997*
*Accepted 22 August 1997*

*Correspondence to:* N Harada

### MATERIALS AND METHODS

### Reagents

Atamestane (1-methyl-androsta-1,4-diene-3, 17-dione), 4-hydroxyandrostenedione, fadrozole (4-(5,6,7,8-tetrahydroimidazo-[1,5a]-pyridine-5-yl)-benzonitrile monohydrochloride), vorozole

**Figure 1** Immunocytochemistry of aromatase in JEG-3 and HepG2 cells treated with aromatase inhibitors. JEG-3 (**A–C**) and HepG2 (**D–F**) cells were cultured in the presence of 10 μM aromatase inhibitors at 37°C for 24 h, and then the immunoreactive aromatase in the control (**A** and **D**), vorozole-treated (**B** and **E**), and atamestane-treated (**C** and **F**) cells was observed by fluorescent microscope. Bars = 20 μm

(6-[(4-chlorophenyl)(1H-1,2,4-triazol-1-yl)methyl]1-methyl-1H-benzotriazole), and pentrozole (5-[cyclopentylidene-(1-imidazolyl)-methyl]-thiophene-2-carbonitrile monohydrochloride), were kindly synthesized and provided by the laboratories of Schering AG (Berlin, Germany). Biotin (long arm) NHS and alkaline phosphatase streptavidin were obtained from Vector Laboratories (Burlingame, CA, USA). Microtitre plates (MaxiSorp) were purchased from Nunc (Roskilde, Denmark).

### Cell culture

Human choriocarcinoma-derived JEG-3 and hepatoma-derived HepG2 cells, obtained from American Type Culture Collection (Bethesda, MD, USA) and Riken Cell Bank (Tsukuba, Japan), respectively, were maintained in minimum essential medium alpha-modification supplemented with 10% fetal calf serum at 37°C in a 95% air/5% carbon dioxide humidified atmosphere. To assess the effects of the inhibitors on aromatase protein, the cells were exposed to 10 μM aromatase inhibitors for 12, 24 or 48 h. The contents of aromatase protein in the cells were determined by ELISA. To confirm inhibition of cellular protein synthesis by cycloheximide, incorporation of L-[4,5-$^{3}$H]leucine into aromatase in JEG-3 cells was examined, as described previously (Harada and Omura, 1983).

### Preparation of total RNA and microsomal fractions from cultured cells

After washing with Dulbecco's phosphate-buffered saline (PBS), cells were scraped from culture dishes and homogenized with 0.25 M sucrose containing 10 mM Tris-HCl (pH 7.5) and 1 mM EDTA. Microsomal fractions were prepared by successive centrifugation (Harada and Omura, 1980). Total RNA fractions were isolated from cells using the Trizol reagent (Gibco-BRL, Gaithersburg, MD, USA) according to the manufacturer's instructions and suspended in 10mM Tris-HCl (pH 7.5) containing 1 mM EDTA and 0.05 units ml$^{-1}$ of ribonuclease inhibitor (Inhibit-ACE; 5Prime-3Prime, Boulder, CO, USA).

### Aromatase immunocytochemical staining

A rabbit polyclonal anti-human aromatase antibody was prepared against the enzyme purified from human placenta (Harada, 1988). This antibody has been confirmed to be monospecific using biochemical and immunological tests (Harada, 1988) and been validated for the detection of aromatase by immunocytochemical staining (Naganuma et al, 1990). Furthermore, the immunospecificity of the antibody was demonstrated by Western blotting analysis of aromatase in JEG-3 cells treated or untreated with aromatase inhibitors, which showed only a single protein band

 © Cancer Research Campaign 1998

**Figure 2** Time-course of increase of aromatase protein in JEG-3 cells treated with various aromatase inhibitors. JEG-3 cells were cultured in the absence (○) or presence of 10 μM 4-OHA (●), atamestane (△), pentrozole (▲), vorozole (□), or fadrozole (■) at 37°C. The cells were collected 12, 24 and 48 h after the addition of aromatase inhibitors, and the contents of aromatase protein in the cells were determined by ELISA. Results are the means ± s.e.m. of three experiments. *$P < 0.05$ compared with the respective control values

**Table 1** Effects of aromatase inhibitors on aromatase protein levels in JEG-3 cells

| Treatment | Aromatase content (ng mg$^{-1}$ of microsomal protein) |
|---|---|
| Before forskolin stimulation | 10.2 ± 0.9 |
| Forskolin stimulation for 48 h | 121.5 ± 5.1 |
| 24 h after removal of forskolin | |
| Control | 55.7 ± 1.7 |
| Atamestane | 99.6 ± 6.9* |
| 4-OHA | 58.9 ± 2.8 |
| Fadrozole | 202.1 ± 9.3* |
| Vorozole | 192.4 ± 7.8* |
| Pentrozole | 196.1 ± 9.1* |

Forskolin was given to JEG-3 cells at 10 μM to induce aromatase. After pretreatment for 48 h, the cells were washed with PBS to completely remove the forskolin and then further cultured for 24 h without forskolin in the presence of 10 μM aromatase inhibitors or in the absence of the inhibitors as a control. Results are the means ± s.e.m. of three experiments. *$P < 0.05$ compared with the control.

with the molecular size corresponding to human aromatase. The immunocytochemical procedures used have been described previously (Hatano et al, 1994). Immunocytochemical staining of aromatase was carried out using rabbit anti-human aromatase and FITC-labelled donkey anti-rabbit immunoglobulin antibodies as primary and secondary antibodies respectively.

### Enzyme-linked immunosorbent assay (ELISA)

The rabbit polyclonal anti-human aromatase antibodies (15 mg) were conjugated with 8 μmol of biotin (long arm) NHS. We first determined optimal conditions for ELISA. High concentrations of detergent and glycerol were found to be significantly inhibitory for the immunological reaction and a high concentration of proteins disturbed the accuracy of quantitation. Microsomal fractions (0.5 mg ml$^{-1}$) were solubilized with 50 mM Tris-HCl (pH 8.0) containing 0.15 M NaCl, 0.1% Tween 20, 0.2% sodium cholate and then centrifuged at 105 000 × $g$ for 90 min to obtain the solubilized supernatants. Microtitre wells were precoated with 2 μg ml$^{-1}$ anti-aromatase antibody at 37°C for 2 h. After washing with PBS, blocking with PBS containing 1% BSA at room temperature for 30 min, and again washing with PBS, 200-μl aliquots of the solubilized supernatants of microsomal fractions were added to the wells and incubated at room temperature for 2 h. Wells for blanks and aromatase standards were also included with the addition of serial dilutions (0–50 ng ml$^{-1}$) of purified human aromatase in place of solubilized supernatant. After washing with PBS, 200 μl of biotin-labelled anti-aromatase antibody (5 μg ml$^{-1}$ in PBS containing 1% BSA) was added to each well, followed by incubation at room temperature for 1 h. After washing with PBS, 200 μl of alkaline phosphatase-streptavidin was added to each well, and incubated for 30 min. After washing with PBS, 200 μl aliquots of 2 mg ml$^{-1}$ *p*-nitrophenylphosphate in 0.1 M diethanolamine (pH 9.8) containing 1 mM magnesium chloride were added to the wells. After further incubation for 40 min in the dark, the reactions were stopped by adding 50 μl of 2 M sodium hydroxide. The absorbance was measured at 405 nm on a Microplate Reader MTP-32 (Corona Electric, Katsuta, Japan). Protein concentrations were determined with a BCA protein assay kit (Pierce, Rockford, IL, USA) using BSA as a standard.

### Quantitative analysis of aromatase mRNA

The aromatase mRNA levels in total RNA fractions were fluorometrically determined by reverse transcription-polymerase chain reaction (RT-PCR) using a fluorescent dye, FAM-labeled primer (Perkin Elmer, Foster City, CA, USA) in the presence of an internal standard RNA, as previously described (Harada et al, 1995; Utsumi et al, 1996). The sequence between PCR primer sites is interrupted by two introns in human aromatase gene. The internal standard RNA was synthesized with T7 RNA polymerase using modified human aromatase cDNA, which was constructed by inserting a 21-bp fragment of *Hae*III-digested λ-DNA between PCR primer sites, as a template. The fluorescent RT-PCR products were analysed on a 2% agarose gel with a Gene Scanner 362 fluorescent fragment analyser (Perkin-Elmer, Foster City, CA, USA). The amount of aromatase mRNA in the total RNA was calculated from the peak areas of the fluorescent products by the internal standard method.

### Statistical analysis

Statistical analysis was performed using one-way analysis of variance (ANOVA), followed by the Scheffe test. A $P$-value $< 0.05$ was considered to be significant.

### RESULTS

### Immunocytochemical analysis in aromatase inhibitor-treated cells

Changes in the protein levels of aromatase in human choriocarcinoma-derived JEG-3 and hepatoma-derived HepG2 cells by

© Cancer Research Campaign 1998

**Table 2** Effects of aromatase inhibitors on the levels of aromatase mRNA in JEG-3 cells

| Treatment | Aromatase mRNA ($10^{-18}$ mol $\mu g^{-1}$ of total RNA) |
|---|---|
| Control (untreated) | 6.07 ± 0.32 |
| Control (forskolin) | 51.72 ± 1.16 |
| Atamestane | 6.31 ± 0.20 |
| 4-OHA | 5.98 ± 0.05 |
| Fadrozole | 6.04 ± 0.15 |
| Vorozole | 5.95 ± 0.19 |
| Pentrozole | 6.14 ± 0.24 |

Forskolin was given to JEG-3 cells at 10 μM to induce aromatase mRNA as a positive control. Results are the means ± s.e.m. of three experiments.

**Table 3** Effects of cycloheximide on the increase of aromatase protein in JEG-3 cells caused by aromatase inhibitors

| Treatment | Aromatase content (ng $mg^{-1}$ of microsomal protein) |
|---|---|
| Control (untreated) | 4.24 ± 0.41 |
| Atamestane | 7.16 ± 0.98 |
| 4-OHA | 4.41 ± 0.31 |
| Fadrozole | 13.57 ± 0.95* |
| Vorozole | 12.73 ± 0.80* |
| Pentrozole | 14.61 ± 1.13* |

JEG-3 cells were cultured in the presence of 20 μg $ml^{-1}$ cycloheximide together with 10 μM aromatase inhibitors at 37°C for 24 h. Results are the means ± s.e.m. of three experiments. *$P < 0.05$ compared with the control.

treatment of aromatase inhibitors were first examined using immunocytochemical staining. As shown in Figure 1, vorozole, a non-steroidal aromatase inhibitor, markedly increased immunoreactive aromatase protein in both JEG-3 (B) and HepG2 (E) cells, compared with untreated controls (A and D). Only a slight increase in aromatase protein was observed with atamestane, a steroidal aromatase inhibitor (C and F). Similar increase in both cells was observed with the other non-steroidal aromatase inhibitors, fadrozole and pentrozole, whereas the steroidal aromatase inhibitor, 4-OHA, hardly gave significant changes in both cells (data not shown).

### Time-dependent changes of aromatase protein levels in aromatase inhibitor-treated JEG-3 cells

To quantitatively investigate the increase in aromatase protein in JEG-3 cells by aromatase inhibitors, an ELISA was developed and checked for its specificity and quantitativeness. As shown in Figure 2, although aromatase protein in untreated control cells remained practically unchanged during the 48-h observation period, all of the non-steroidal aromatase inhibitors, fadrozole, vorozole and pentrozole, caused a time-dependent increase in aromatase protein to about fourfold the control level after 24 h. In contrast, although a time-dependent increase in aromatase protein was also observed with the steroidal aromatase inhibitor atamestane it was to a much lesser extent, and no significant effects of the other steroidal aromatase inhibitor, 4-OHA, were noted.

### Effects of aromatase inhibitors on decrease in aromatase protein levels after forskolin induction in JEG-3 cells

The effects of aromatase inhibitors on protein levels of aromatase in JEG-3 cells were further investigated after the induction of aromatase by forskolin. Aromatase was elevated about tenfold (121.5 ± 5.1 ng $mg^{-1}$ microsomal protein) 48 h after the treatment of forskolin. As shown in Table 1, the induced level was decreased to about half 24 h after the removal of forskolin. This reduction was not affected by 4-hydroxyandrostenedione treatment but was prevented by atamestane. In contrast, the addition of the non-steroidal aromatase inhibitors to the cells caused significant increases in aromatase protein in spite of the absence of the inducer.

### Effects of aromatase inhibitors on the levels of aromatase mRNA in JEG-3 cells

The levels of aromatase mRNA in JEG-3 cells treated with 10 μM aromatase inhibitors for 24 h were fluorometrically determined by a quantitative RT-PCR method using an internal standard RNA and a fluorescent dye-labelled primer. Table 2 summarizes the results. The level of aromatase mRNA was increased approximately ninefold in the cells treated with forskolin as a positive control, whereas no significant intergroup differences were observed in the cells treated with aromatase inhibitors, indicating that all of the inhibitors have no effects on the levels of aromatase mRNA in the cells.

### Effects of cycloheximide on the inhibitor-associated increase of aromatase protein in JEG-3 cells

The results obtained in this study indicated that the higher levels of aromatase protein in JEG-3 cells treated with aromatase inhibitors was not due to increased mRNA levels, but rather to increased translation or decreased degradation. To assess the first of these possibilities, cycloheximide was simultaneously added as an inhibitor of protein synthesis with aromatase inhibitors, and changes in aromatase protein were again observed after 24 h. As shown in Table 3, the levels of aromatase protein in the cells treated with cycloheximide were all decreased compared with the values without cycloheximide (Figure 2). However, all the non-steroidal aromatase inhibitors still caused an approximately three-fold increase, and atamestane also exerted some apparent effect. 4-Hydroxyandrostenedione, in contrast, did not cause any change in the level of aromatase protein. As a positive control, [$^3$H]leucine incorporation into aromatase in the cells was also examined. It was inhibited more than 95% by cycloheximide, indicating effective suppression of protein synthesis in the cells by this dose of the drug (data not shown). As essentially the same pattern of increase in aromatase protein by aromatase inhibitors was observed with and without cycloheximide, the results indicate that the elevation of aromatase protein associated with aromatase inhibitors is mediated by stabilization of the enzyme and prevention of protein degradation.

### DISCUSSION

In the present study, non-steroidal aromatase inhibitors, fadrozole, vorozole, and pentrozole, clearly increased the immunoreactive aromatase protein in cultured JEG-3 and HepG2 cells. Time

© Cancer Research Campaign 1998

dependence of the inhibitor influence was further demonstrated by quantitative analysis using ELISA. The rapid degradation of forskolin-induced aromatase followed by removal of the inducer also appeared to be prevented by most of the inhibitors. Presumably, they stabilized newly synthesized aromatase as well as the forskolin-induced enzyme and, consequently, protein levels in the cells were enhanced above the forskolin-stimulated level. Additional evidence that the decrease in the turnover of enzyme protein was responsible for the observed increase was provided by the lack of apparent change in aromatase mRNA level and by the fact that cycloheximide did not cause significant alteration of the pattern of elevation. Thus, the results strongly suggest that aromatase inhibitors exert their action on aromatase protein through protection against protein degradation, rather than through increase in transcription or translation, or mRNA stabilization. A similar increase in aromatase protein in JEG-3 cells treated with aminoglutethimide and fadrozole was also observed in another laboratory (W Yue and AMH Brodie, personal communication). Furthermore, Miller and Mullen (1993) showed that aromatase activities in the tumours of breast cancer patients after aromatase inhibitor (aminoglutethimide) treatment were significantly higher than those of the same patients before the treatment. This result supports the clinical significance of our observation.

In the present study, we used aromatase inhibitors at the concentration of 10 μM. This is quite high compared with the minimum concentration required to inhibit aromatase and also seems to be high compared with those found in patients after clinical administration of the inhibitors. Therefore, we performed additional experiments to examine the effects of 1 and 0.1 μM inhibitor concentrations on the levels of aromatase in JEG-3 cells and obtained essentially similar results. The observed higher levels of aromatase protein in the cells indicate that clinical doses of the inhibitors might also generate higher levels of aromatase in patients (data not shown). Increased dosage of such inhibitors might be necessary to maintain complete suppression of cancer cells, which otherwise might escape from aromatase inhibitor control. Recently, anti-tumour hormone therapy with aromatase inhibitors has been introduced for oestrogen-dependent cancers, especially breast cancers. The data from clinical trials have suggested that this may be an effective therapy without serious harmful effects. However, it appears to be necessary to re-examine further the possibility that aromatase inhibitor therapy could induce a situation in which higher doses of inhibitors might be required.

There are several possible explanations for the distinct differences in the effects of the various aromatase inhibitors used. First, the capacities of aromatase inhibitors to increase aromatase protein in cells may reflect their binding affinities ($K_i$-values) for aromatase. The $K_i$-values of 4-OHA and atamestane are reported to be about 250 nM (Henderson et al, 1986), whereas those of fadrozole and vorozole are 1.6 (Steele et al, 1987) and 0.7 nM (Vanden Bossche et al, 1990) respectively. Judging from this difference, non-steroidal inhibitors would be expected to be more tightly associated with the aromatase molecules in stable complexes that may be more resistant to proteolytic cleavage. Second, steroidal aromatase inhibitors, 4-OHA and atamestane, were known to be mechanism-based inhibitors or suicide inhibitors (Brodie et al, 1981; Henderson et al, 1986). They promote time-dependent inactivation of aromatase through production of a reactive intermediate by the catalytically active enzyme, and probably time-dependent degradation due to increasing sensitivity of the inactive form to proteolytic cleavage. It is likely that 4-OHA is a stronger mechanism-based inhibitor than atamestane as their inactivation rates of aromatase are reported to be $4.5 \times 10^{-3}$ (Brodie et al, 1981) and $1.8 \times 10^{-3}$ $s^{-1}$ (Henderson et al, 1986) respectively. Consequently, this may be the reason why 4-OHA did not cause an appreciable increase in aromatase protein, in contrast to atamestane.

Aromatase inhibitors and substrates are thought to competitively bind to the same binding sites within aromatase molecules and to form conformationally tight complexes, so that they could be expected to be resistant to proteolytic degradation. Stabilization and increased content caused by substrates or inhibitors has been found for many enzymes; for example the cellular levels of arginase (Schimke, 1964) and tryptophan pyrrolase (Schimke et al, 1965) in rat liver are known to become elevated because of decreased protein degradation dependent on the substrates arginine and tryptophan. Furthermore, steroidogenic cytochrome P-$450_{11\beta}$ and aromatase could be successfully purified in the presence of deoxycorticosterone (Takemori et al, 1975) and testosterone (Harada, 1988) or androstenedione (Tan and Muto, 1986), used as substrate stabilizers to prevent inactivation of the enzyme. Recently, immunoreactive aromatase in quail brain was found to be increased by the specific inhibitors fadrozole and vorozole (Foidart et al, 1994). These results suggest that tight aromatase/inhibitor complexes are also relatively resistant to proteolytic degradation and, consequently, cause higher levels of aromatase protein.

For the purposes of the present study, we developed ELISA for quantitative analysis of aromatase protein in the cells. Kitawaki et al (1989) previously introduced a similar method for quantitation of catalytically active aromatase in human placenta, but the content of aromatase in microsomes of JEG-3 cells is quite low (about 10 ng $mg^{-1}$ of protein) compared with the placenta case (about 15 μg $mg^{-1}$ of protein). Therefore, we solubilized microsomes with low concentrations of Tween 20 and sodium cholate and performed ELISA in the absence of glycerol to avoid any unnecessary interference with the immunological reaction. Under these conditions, protein recovery was improved and accurate quantitation could be confirmed. The aromatase was the more stable P-420 form (Omura and Sato, 1964) that is catalytically inactive.

The results obtained in this study support a conclusion that blockage of aromatase degradation by inhibitors results in marked increase in immunoreactive protein in cells in culture.

### ACKNOWLEDGEMENTS

This work was supported in part by Grants-in-Aid for Research from Fujita Health University and Grants-in-Aid for Scientific Research from the Ministry of Education, Science, and Culture of Japan. We thank Dr Malcolm Moore for correcting the English.

### REFERENCES

Abul-Hajj YJ, Iverson R and Kiang DT (1979) Aromatization of androgens by human breast cancer. *Steroids* **33**: 205–222

Amarneh B, Corbin CJ, Peterson JA, Simpson ER and Graham LS (1993) Functional domains of human aromatase cytochrome P450 characterized by linear alignment and site-directed mutagenesis. *Mol Endocrinol* **7**: 1617–1624

Brodie AM (1994) Aromatase inhibitors in the treatment of breast cancer. *J Steroid Biochem Mol Biol* **49**: 281–287

© Cancer Research Campaign 1998

Brodie AMH, Garrett W, Hendrickson JR, Tsai-Morris C-H, Marcotte CH and Robinson CH (1981) Inactivation of aromatase *in vitro* by 4-hydroxy-4-androstene-3,17-dione and 4-acetoxy-4-androstene-3,17-dione and sustained effects *in vivo*. *Steroids* **38**: 693–702

Bulun SE, Price TM, Aitken J, Mahendroo MS and Simpson ER (1993) A link between breast cancer and local estrogen biosynthesis suggested by quantification of breast adipose tissue aromatase cytochrome P450 transcripts using competitive polymerase chain reaction after reverse transcription. *J Clin Endocrinol Metab* **77**: 1622–1628

Chen S and Zhou D (1992) Functional domains of aromatase cytochrome P450 inferred from comparative analyses of amino acid sequences and substantiated by site-directed mutagenesis experiments. *J Biol Chem* **267**: 22587–22594

Esteban JM, Warsi Z, Haniu M, Hall P, Shively JE and Chen S (1992) Detection of intratumoral aromatase in breast carcinomas. An immunohistochemical study with clinicopathologic correlation. *Am J Pathol* **140**: 337–343

Foidart A, Harada N and Balthazart J (1994) Effects of steroidal and non steroidal aromatase inhibitors on sexual behavior and aromatase-immunoreactive cells and fibers in the quail brain. *Brain Res* **657**: 105–123

Graham-Lorence S, Khalil MW, Lorence MC, Mendelson CR and Simpson ER (1991) Structure–function relationships of human aromatase cytochrome P-450 using molecular modeling and site-directed mutagenesis. *J Biol Chem* **266**: 11939–11946

Graham-Lorence S, Amarneh B, White RE, Peterson JA and Simpson ER (1995) A three-dimensional model of aromatase cytochrome P450. *Protein Sci* **4**: 1065–1080

Harada N (1988) Novel properties of human placental aromatase as cytochrome P-450; purification and characterization of a unique form of aromatase. *J Biochem* **103**: 106–113

Harada N and Omura T (1980) Participation of cytochrome P-450 in the reduction of nitro compounds by rat liver microsomes. *J Biochem* **87**: 1539–1554

Harada N and Omura T (1983) Phenobarbital- and 3-methylcholanthrene-induced synthesis of two different molecular species of microsomal cytochrome P-450 in rat liver. *J Biochem* **93**: 1361–1373

Harada N, Utsumi T and Takagi Y (1995) Molecular and epidemiological analyses of abnormal expression of aromatase in breast cancer. *Pharmacogenetics* **5**: 59–64

Hatano O, Takayama K, Imai T, Waterman MR, Takakusu A, Omura T and Morohashi K (1994) Sex-dependent expression of a transcription factor, Ad4BP, regulating steroidogenic P-450 genes in the gonads during prenatal and postnatal rat development. *Development* **120**: 2787–2797

Henderson D, Norbisrath G and Kerb U (1986) 1-Methyl-1, 4-androstadiene-3,17-dione (SH 489): Characterization of an irreversible inhibitor of estrogen biosynthesis. *J Steroid Biochem Mol Biol* **24**: 303–306

Kitawaki J, Yoshida N and Osawa Y (1989) An enzyme-linked immunosorbent assay for quantitation of aromatase cytochrome P-450. *Endocrinology* **124**: 1417–1423

Lu Q, Nakmura J, Savinov A, Yue W, Weisz J, Dabbs DJ, Wolz G and Brodie A (1996) Expression of aromatase protein and messenger ribonucleic acid in tumor epithelial cells and evidence of functional significance of locally produced estrogen in human breast cancers. *Endocrinology* **137**: 3061–3068

Marsh DA, Brodie HJ, Garrett WM, Tsai-Morris CH and Brodie AMH (1985) Aromatase inhibitors – synthesis and biological activity of androstenedione derivatives. *J Med Chem* **28**: 788–795

Miller WR and Mullen P (1993) Factors influencing aromatase activity in the breast. *J Steroid Biochem Mol Biol* **44**: 597–604

Miller WR and O'Neill J (1987) The importance of local synthesis of estrogen within the breast. *Steroids* **50**: 537–548

Naganuma H, Ohtani H, Harada N and Nagura H (1990) Immunoelectron microscopic localization of aromatase in human placenta and ovary using microwave fixation. *J Histochem Cytochem* **38**: 1427–1432

Noble LS, Simpson ER, Johns A and Bulun SE (1996) Aromatase expression in endometriosis. *J Clin Endocrinol Metab* **81**: 174–179

Omura T and Sato R (1964) The carbon monoxide-binding pigment of liver microsomes. *J Biol Chem* **239**: 2379–2385

Poulos TL, Finzel BC, Gunsalus IC, Wagner GC and Kraut J (1985) The 2.6 A crystal structure of *Pseudomonas putida* cytochrome P-450. *J Biol Chem* **260**: 16122–16130

Ravichandran KG, Boodupalli SS, Haseman CA, Peterson JA and Deisenhofer J (1993) Crystal structure of hemoprotein domain of P450BM-3, a prototype of microsomal P450s. *Science* **261**: 731–736

Santen RJ and Misbin RI (1981) Aminoglutethimide: review of pharmacology and clinical uses. *Pharmacotherapy* **1**: 95–120

Santen RJ, Martel J, Hoagland M, Naftolin F, Roa L, Harada N, Hafer L, Zaino R and Santner SJ (1994) Stromal spindle cells contain aromatase in human breast tumors. *J Clin Endocrinol Metab* **79**: 627–632

Santner SJ, Feil PD and Santen RJ (1984) In situ estrogen production via the estrogen sulfatase pathway in breast tumors: relative importance versus the aromatase pathway. *J Clin Endocrinol Metab* **59**: 29–33

Sasano H, Nagura H, Harada N, Goukon Y and Kimura M (1994) Immunolocalization of aromatase and other steroidogenic enzymes in human breast disorders. *Hum Pathol* **25**: 530–535

Schimke RT (1964) The importance of both synthesis and degradation in the control of arginase levels in rat liver. *J Biol Chem* **239**: 3808–3817

Schimke RT, Sweeney EW and Berlin CM (1965) The roles of synthesis and degradation in the control of rat liver tryptophan pyrrolase. *J Biol Chem* **240**: 322–331

Steele RE, Mellor LB, Sawyer WK, Wasvary JM and Brown RE (1987) *In vitro* and *in vivo* studies demonstrating potent and selective estrogen inhibition with the nonsteroidal aromatase inhibitor CGS16949A. *Steroids* **50**: 147–161

Takemori S, Sato H, Gomi T, Suhara K and Katagiri M (1975) Purification and properties of cytochrome P-450 11β from adrenocortical mitochondria. *Biochem Biophys Res Commun* **67**: 1151–1157

Tan J and Muto N (1986) Purification and reconstitution properties of human placental aromatase; a cytochrome P-450-type monooxygenase. *Eur J Biochem* **156**: 243–250

Utsumi T, Harada N, Maruta M and Takagi Y (1996) Presence of alternatively spliced transcripts of aromatase gene in human breast cancer. *J Clin Endocrinol Metab* **81**: 2344–2349

Vanden Bossche H, Willemsens G, Roels I, Bellens D, Moereels H, Coene MC, LeJeune L, Lauwers W and Janssen PAJ (1990) R76713 and enantiomers: selective, nonsteroidal inhibitors of the cytochrome P450-dependent estrogen synthesis. *Biochem Pharmacol* **40**: 1707–1718

Zhou DJ, Pompon D and Chen SA (1991) Structure–function studies of human aromatase by site-directed mutagenesis: kinetic properties of mutants Pro-308–Phe, Tyr-361–Phe, Tyr-361–Leu, and Phe-406–Arg. *Proc Natl Acad Sci USA* **88**: 410–414

© Cancer Research Campaign 1998